# Self-reported COVID-19 among physicians: An Egyptian online study during the pandemic

**DOI:** 10.12688/f1000research.53931.1

**Published:** 2021-08-10

**Authors:** Hala Samir Abou-ElWafa, Abdel-Hady El-Gilany, Ahmed A. Albadry

**Affiliations:** 1 MD of Industrial Medicine and Occupational Health‎, Public Health & Community Medicine Department, Faculty of Medicine, Mansoura University., Mansoura, Egypt; 2 ‎MD of Public Health and Preventive Medicine, Public Health & Community Medicine ‎Department, Faculty of Medicine, Mansoura University., Mansoura, Egypt

**Keywords:** COVID-19, physicians, pandemic, incidence, Egypt, frontline, PPE

## Abstract

Abstract:

** Background:** COVID-19 causes a critical occupational risk to frontline healthcare workers (HCWs) who respond to the pandemic, as they are placed in environments with an increased risk of infection exposure. It is a public health priority to understand how transmission occurs to protect this vulnerable group of HCWs. This study was conducted to estimate the incidence of self-reported COVID-19 infection among physicians and its possible associated factors. **Methods:** An online national survey using Survey Monkey was initiated to collect sociodemographic e.g. age and sex, occupational e.g. place and duration of work, and clinical data e.g. COVID symptoms and laboratory investigations, and to describe affected physicians' diagnoses. Results: The self-reported incidence of COVID-19 infection was found to be 65.4% among studied physicians. The significant independent predictors of COVID-19 infection were  smoking, working as a frontline physician, having contact with a COVID-19 case, and working for less than ten years [ARR (95% CI): 3.0(1.6-5.7), 2.3(1.4-3.8), 2.1(1.2-3.6), and 1.8(1.2-2.9); respectively]. **Conclusions:** The incidence of COVID-19 infection among Egyptian physicians is relatively high. Smoking, being a frontline physician, having contact with a COVID-19 case, and working for less than 10 years are all factors associated with an increased risk of infection. There should be strict application of preventive measures, periodic screening for COVID-19 for early detection and isolation of infected HCWs together with effective vaccination.

## Introduction

COVID-19, has become a universal threat to public health ^1^. Since the beginning of the COVID-19 pandemic, healthcare workers have exhibited tremendous strength and professional loyalty despite the risk of infection and spreading the infection to others ^2^.

During the initial wave of the COVID-19 pandemic, overstrained healthcare systems in severely affected countries left healthcare workers fighting with prolonged work hours, intense emotional stress, and fatigue. Speedily declining resources, lockdowns, and a high demand for personal protective equipment (PPE) led to shortages ^3^. Healthcare workers frequently had to care for patients with confirmed or suspected COVID-19 infections with insufficient PPE or improper training. These conditions contributed to a heightened risk to healthcare workforces of contracting the infection throughout the pandemic's early stage ^4– 6^.

Most studies reported enhanced risks for health professionals who care for COVID-19 patients ^5, 7, 8^. The ultimate threat to healthcare professionals may be their contact with colleagues or patients in the early stages of unpredicted infections when viral loads are at a high-level ^9^.

Based on previous respiratory virus experience, consistent PPE use is essential for managing nosocomial transmission ^10^. Strategies from the USA and the UK recommended that healthcare workers wear a surgical mask when providing care for individuals with COVID-19 ^11^. However, worldwide deficiencies of masks, face shields, respirators, and gowns, triggered by heavy needs and supply series disturbances, have directed efforts to save PPE throughout prolonged usage or reuse, Disinfection procedures have also been employed, but there is a lack of adequate agreement on best practice ^12– 14^.

According to the published articles, many healthcare professionals have been infected with COVID-19 internationally and have even died as a result ^15– 17^. Consequently, adequate protection for HCWs is of utmost importance ^18^. To the best of our knowledge, there is currently no published work about the incidence of COVID-19 infection among physicians in Egypt. Therefore, this study was performed to estimate the annual incidence, a year before data collection, of self-reported infection among physicians and its possible associated factors including sociodemographic, occupational, medical and preventative factors.

## Methods

### Study design

Descriptive longitudinal study with an analytical component.

A national online survey was carried out among Egyptian physicians from December 26, 2020, to January 16, 2021. 

### Target population and sample size calculation

Egyptian physicians working anywhere in Egypt that have been on duty for one year or more. The sample size was calculated online using the Open Epi Program., Version 3.01. A pilot study on 100 physicians discovered that 70% reported being infected with COVID-19, whether possible, probable, or confirmed. With a 95% confidence level and 5% precision, the sample size was 323 physicians. 

### Study tool

A survey form, developed by the authors, was used to collect the following data: socio-demographics e.g. sex, age, smoking; work profile, medical history; preventive measures at the workplace, e.g. according to the Egyptian guidelines, PPE use, hand sanitization, and diagnostic criteria of cases ^19^. Participants selected the signs and symptoms they experienced during the past year from a list of all possible COVID-19 manifestations. The form allowed participants to choose a final diagnosis, whether suspected, probable, or a confirmed case, guided by their answers on the relevant criteria. Participants were encouraged to respond to understand the problem's magnitude and its risk factors. 

Participants were enrolled via social media (e.g. Facebook and Whatsapp) and invited by the researchers to respond voluntarily to the survey. At enrolment, participants gave consent for the information they provided to be used for research. Consideration of privacy was guaranteed as data were collected anonymously. 

Data were collected using Survey Monkey, which was launched online, and the survey was open until the required sample size was reached. A copy of the survey can be found here ( https://doi.org/10.7910/DVN/BVSLG2)

### Ethical approval

The Institutional Research Board (IRB) Approval at Faculty of Medicine, Mansoura University, was obtained. Registration number (R.20.11.1078)

### Informed consent

Participants were asked to voluntarily participate in the study to ensure confidentiality and anonymity of data; the online survey include the first question about informed consent to participate in the research and publication of these results; the question was tick box, if not ticked the survey end automatically.

### Data analysis

Data were entered/ and statistically analyzed, applying the Statistical Package for Social Sciences (SPSS) version 23. The accompanying dataset is openly available on Harvard Dataverse ( https://doi.org/10.7910/DVN/BVSLG2). Categorical variables were portrayed as numbers and percentages. The χ ^2^ test was used to check the significance in bivariate analysis, and crude risk ratios (CRR) and 95% CI were assessed. Variables significantly associated with COVID-19 infection in bivariate analysis were entered into a multivariate logistic regression model using the forward Wald method. Adjusted risk ratios (ARR) and their 95% CI were calculated. "p-value ≤0.05" was considered statistically significant. Participants had to complete the whole survey to get their final diagnosis, so missing data were not possible.

## Results

Table 1 reveals that 65.4% of physicians reported COVID-19 infection. About three-quarters of physicians under 35 years old were infected compared to 57.7% of those aged 35 years and older, with a statistically significant difference between them (p= 0.007). A higher percentage of frontline physicians were infected than those not on the front line with a statistically significant difference (p=0.001). Having contact with a COVID-19 case showed a statistically significant difference (p=0.001), and most smokers (82.3%) and those working for less than ten years (73.1%) were significantly infected.

**Table 1.** Incidence of self-reported COVID-19 and its associated sociodemographic and work factors.

	Total	Infected	P-value	Crude RR (95% CI)
Overall	355	232(65.4)		
Age: ≤35 years >35 years	184 171	133(72.3) 99(57.7)	0.007	1.2(1.1-1.5) r
Sex: Male Female	210 145	139(66.2) 93(64.1)	0.7	1.0(0.7-1.3) r
Marital status: Unmarried * Married	66 289	49(74.2) 183(63.3)	0.1	1.2(0.98-1.4) r
Job title: Resident Specialist Consultant/faculty	94 175 86	68(72.3) 109(62.3) 55(64.0)	0.2 0.8	1.1(0.9-1.4) 0.97(0.8-1.2) r
Education: M.B-B.Ch/Diploma M. Sc. Others **	77 167 111	55(71.7) 104(67.3) 73(65.8)	0.4 0.6	1.1(0.9-1.3) 0.9(0.8-1.1) r
Specialty: Medical specialties Critical care/emergency Surgical specialties Others ***	190 33 88 44	128(67.4) 21(63.6) 53(60.2) 44(68.2)	0.9 0.7 0.4	0.99(0.8-1.2) 0.9(0.7-1.3) 0.9(0.7-1.2) r
Workplace: University hospital Governmental hospital Others # More than one place	71 142 73 69	46(64.8) 95(66.9) 45(51.6) 46(66.7)	0.8 0.97 0.5	0.97(0.8-1.2) 1.0(0.8-1.2) 0.9(0.7-1.2) r
Work schedule: Regular Shift	198 157	122(61.6) 110(70.1)	0.1	0.8(0.7-1.0) r
Work duration: ≤ 10 years >10 years	197 158	144(73.1) 88(55.7)	0.001	1.3(1.1-1.5) r
Frontline: Yes No	263 92	185(70.3) 47(51.1)	0.001	1.4(1.1-1.7) r
Contact with a COVID-19 case: Yes No	279 76	195(69.9) 37(48.7)	0.001	1.4(1.1-1.8) r
Tobacco smoking: Yes No	79 276	65(82.3) 167(60.5)	≤0.001	1.4(1.2-1.6) r
Chronic disease: Yes ## No	124231	77(62.1)155(67.1)	0.3	0.9(0.8-1.1) r

*Single, widow & divorced **M.D., Ph. D, fellowship & board ***General practitioner, family medicine, public health, laboratory medicine #Primary health care, Family practice, private, insurance ##Obesity, hypertension, diabetes, cardiac diseases RR=risk ratio, CI=Confidence Interval r=reference category

The incidence of infection does not vary significantly with the use of PPE and hand sanitizers (see Table 2).

**Table 2.** Incidence of self-reported COVID-19 according to use of PPE and hand sanitizers.

	Total	Infected	P-value	Crude RR (95% CI)
Overall	355	232(65.4)		
Facemask use: Yes No	333 22	219(65.8) 13(59.1)	0.5	1.1(0.8-1.6) r
N95 respirator use: Yes No	56 299	38(67.9) 194(64.9)	0.7	1.0(0.9-1.3) r
Face shield use: Yes No	108 247	70(64.8) 162(65.6)	0.9	0.99(0.8-1.2) r
Goggle use: Yes No	40 315	24(60.0) 208(66.0)	0.5	0.9(0.7-1.2) r
Head cap use: Yes No	75 280	53(70.7) 179(63.9)	0.3	1.1(0.9-1.1) r
Gloves use: Yes No	262 93	174(66.4) 58(62.4)	0.5	1.1(0.9-1.3) r
Gown use: Yes No	141 214	94(66.7) 138(64.5)	0.7	1.0(0.9-1.2) r
Coverall use: Yes No	51 304	37(72.5) 195(64.1)	0.2	1.1(0.9-1.4) r
Shoe cover use: Yes No	49 306	31(63.3) 201(65.7)	0.7	0.96(0.8-1.2) r
Sanitizer use: Yes No	309 46	200(64.7) 32(69.6)	0.5	0.9(0.8-1.1) r

RR=risk ratio, CI=Confidence Interval, r=reference category

Table 3 demonstrates that the significant independent predictors of COVID-19 infection were smoking, working as a frontline physician, having contact with a COVID-19 case, and working for less than ten years [ARR (95% CI): 3.0(1.6-5.7), 2.3(1.4-3.8), 2.1(1.2-3.6), and 1.8(1.2-2.9); respectively].

**Table 3.** Logistic regression analysis of independent predictors of self-reported COVID-19 infection.

	β	P-value	ARR (95%CI)
Smoking: Yes No	1.1 -	0.001	3.0(1.6-5.7) r
Frontline: Yes No	0.8 -	0.002	2.3(1.4-3.8) r
Contact with a COVID-19 case: Yes No	0.7 -	0.009	2.1(1.2-3.6) r
Work duration: ≤ 10 years >10 years	0.6 -	0.01	1.8(1.2-2.9) r
Constant Model c ^2^ % correctly predicted Hosmer & Lemeshow test	-1.04 41.5, ≤.0.001 69.6% c ^2^=2.1,P=0.91		

ARR=adjusted risk ratio, CI=Confidence Interval, r=reference category

Most of the self-reported COVID-19 cases (about 71%) were probable cases, and confirmed cases represented 23%. A tiny percentage of cases were asymptomatic (2.6%).

 Among symptomatic cases, the most frequent symptoms were fatigue and a sore throat (about 75% each), and the least frequent was mental confusion (7.8%). The health facility was the most frequent possible source of infection (about half the cases), while the least was the general population source (19.4%) (see Table 4).

**Table 4.** Self-reported incidence and clinical features of COVID-19 cases.

	N(%)
**Diagnostic category:** Suspected Probable Confirmed	15(6.5) 164(70.7) 53(22.8)
Presenting features of self-reported cases: Asymptomatic Symptomatic: Fatigue/general weakness Sore throat Headache Myalgia Respiratory symptoms * Diarrhea Loss of smell &/or taste Fever Nausea & vomiting Mental confusion/dullness Positive chest CT	6(2.6) 172(74.1) 167(72.0) 159(68.5) 144(62.1) 112(48.3) 101(43.5) 92(39.7) 87(37.5) 52(22.4) 18(7.8) 189(81.5)
**The possible source of infection#:** Health facility General population Unknown	110(47.4) 45(19.4) 77(33.2)

*cough, expectoration, chest tightness, dyspnea

## Discussion

Since its beginning2, COVID-19 has become global health threat. ^5^ Healthcare workers are exposed to the infection to a more considerable extent than other members of society and may be judged to be at an elevated risk of disease ^20^. Throughout the crisis, they were continually caring for patients with confirmed or suspected COVID-19 infection with improper training and/or inadequate protective equipment ^4– 6^. 

This study showed that 65.4% of physicians reported having had a COVID-19 infection. This percentage is much higher than the rates reported in other countries. In Brazil, 42.37% of HCWs with symptoms tested positive for COVID-19. In Brazil, over the ten-week study period, the rate of positive testing ranged from 22.2% in the second week to 55.9% in the sixth week, then plateaued to 38–46% ^21^. However, much lower percentages of HCWs who had COVID-19 during the study period were reported in China (33.62%) ^18^ and, in Spain, (11.1%). In Italy, 20% of responding HCWs were infected ^22^. In France, the incidence of COVID-19 positivity was 35% among symptomatic HCWs who were tested ^23^.

A wide variation was found in the prevalence of COVID-19 infection, which fluctuated from 0.4% in Spain ^24^ to 57.06% in New York City ^25^. In Seattle, Washington, the prevalence of COVID-19 amongst frontline HCWs was (5.2%) ^26^.

The proportion of COVID-19 infection also showed a broad variability. In the early published studies from Wuhan, China, 29% of the cases were HCWs ^17^. A much lower proportion of HCWs (3.8%) was found in another study in China ^27^. A slightly higher frequency was reported in Spain, where the ratio of physicians was 13% ^22^. A much higher frequency was reported in another study from Spain, showing that 22% of COVID-19 cases have been amongst HCWs ^22^. In a meta-analysis and systematic review of the prevalence of COVID-19 in HCWs, among HCWs with positive results, 25% were physicians ^28^. 

The high incidence reported in the current study could be attributed to the self-reported diagnosis that includes all probable, possible, and confirmed infections. Other contributing factors include work overload and shortage of PPE, mainly when dealing with undiagnosed cases, extended use or reuse, or suboptimal use (about two-thirds only of studied physicians used PPE and hand sanitizers) and HCW to HCW transmission may play a role. The environmental factors could contribute to this high incidence as hospital air, surfaces, and devices could be contaminated by aerosol from infected patients. Dual sources of infection could play a role as 47.4% of infected COVID-19 cases and 19.4% of them reported that health facilities and the general community were the possible sources of infection, respectively. About one-third of physicians (33%) were unable to predict the source of infection as asymptomatic carriers, and silent spreaders play an essential role in COVID-19 transmission, which may be among infected work colleagues, patients, household contacts, relatives, or friends.

The present study showed that smoking was found to be the most important independent predictor of COVID-19 infection [ARR (95% CI): 3.0 (1.6-5.7)]. For COVID-19, data are limited; one review didn't comment on smoking as a risk factor associated with COVID-19 infection but reported an elevated risk of intense disease [relative risk (R.R.) 1.4 (95% CI 0.98–2)] and the requirement for mechanical ventilation or the infection resulting in death [RR 2.4 (1.43–4.04)] for current smokers ^29^. A meta-analysis included 16 studies and revealed that smoking history and active smoking considerably increased the risk for severe COVID-19 ^30^. However, a prior study has reported a non-significant correlation between smoking history and COVID-19 severity ^31^. 

There are several ways tobacco smoking might increase the risk of COVID-19 infection and result in worse symptoms for COVID-19. There are the general effects of smoking on host defenses and increased inflammation reactions among smokers, and the specific influences on the ACE2 receptor. Moreover, smoking increases the chronic disease frequently associated with elevated risk for COVID-19 sequalae bad outcomes: coronary heart disease, chronic obstructive pulmonary disease, and type 2 diabetes mellitus. Intensified risk for more severe COVID-19 might be arbitrated through chronic diseases attributed to smoking ^32^. 

The present study showed that being a frontline physician was found to be a predictor of COVID-19 infection [ARR (95% CI): 2.3(1.4-3.8)]. This correlation agrees with results from China, where 23.6% of physicians were diagnosed with COVID-19; amongst them, 35.3% were frontlines first-lines, 21.5% non-first lines. Frontline HCWs may have a higher chance of getting an infection due to close patient contact ^33^. COVID-19 infection prevalence was 2747 cases per 100,000 among frontline HCWs in the study carried out in the UK and USA using self-reported data. Frontline HCWs had a twelvefold increase in the risk of testing positive with multivariate adjustment (adjusted HR 11.61, 95% CI 10.93–12.33) ^5^.

The present study showed that having contact with a COVID-19 case was found to be a predictor of COVID-19 infection [ARR (95% CI): 2.1(1.2-3.6)]. Similarly, in China, about 60% of the infected HCWs attributed their infection to contact with later diagnosed patients with COVID-19 ^33^.

Duration of employment Working for less than ten years was found to be an independent predictor of COVID-19 infection [ARR (95% CI): 1.8(1.2-2.9)] in the present study. Similarly, in China, being younger than 45 years old was correlated with an enhanced risk of getting infected (IRR, 1.9; 95%CI, 1.3-3; p = 0.002) ^33^. This study reported that about three-quarters of physicians below 35 years old were infected compared to 57.7% of those above 35 years, with a statistically significant difference between them. Correspondingly, in a Chinese study, about 71% of the infected HCWs were below 45 years old ^33^. The high incidence in this study could be attributed to the younger age of infected doctors (72.3% were <35 years), their inadequate experience (73.1% worked <10 years) and being resident physicians (72.3%) compared to specialists and consultants/faculty members. Also, younger doctors are in more frequent and prolonged contact with patients and their relatives, which increases their chance of contracting the infection as juniors usually carry most of the workload to gain more experience.

Our findings showed that a high percentage of infection was found among doctors working in governmental hospitals and those working in more than one hospital at the same time. In China, (89.26%) of infected HCWs were general hospital staff, then specialized hospital staff (5.70%), and community hospitals staff (5.05%). The case infection rate (CIR) of community hospitals was markedly lower than the general hospitals (p<0.001 and p<0.001; respectively), while the CIR of community hospitals was lower than specialized hospitals (p<0.001) ^18^. Another study from China showed that working in clinical departments other than fever clinics or wards was correlated with a high risk of getting infected (IRR 3.1; 95%CI, 1.8-5.2, p< 0.001) ^33^.

A non-statistically significant difference was found in infection rates of males and females in this study. Similarly, in China, a non-significant difference was found in the CIR of HCWs, including doctors by gender (p = 0.591) ^18^. Both sexes are exposed to the same potential risks of infection in both the hospital care settings and the community.

Department of critical care/emergency specialty did not show a significantly greater incidence of COVID-19 among doctors. Similarly, in the UK, working in ICUs intensive care units were not linked with higher infection risk, probably related to the protection provided by advanced protection by PPE or declining infectivity occurring in subsequent stages of the disease, even amongst seriously ill patients ^7^.

The incidence of infection in this study does not vary significantly with PPE and hand sanitizers. Similarly, in New Jersey, reported PPE use was positively correlated with the number of confirmed or suspected COVID-19 patients, and HCWs who stated less usage of protective equipment did not seem to be at an increased risk of infection, proposing that use of protective measures was comparative to perceived threats of getting an infection ^34^. Notably, a study of healthcare workers in Wuhan, China, throughout the peak of the pandemic revealed that universal PPE use and other protective measures were extraordinarily successful and entirely protected healthcare workers, evidenced by the negativity of both virus and antibody ^35^. Likewise, in Wuhan, China, utilizing medical masks was significantly associated with an elevated risk of COVID-19 in HCWs than using N95 respirators, even with substantially higher exposure to infected patients among the last group ^36^.

The health facility (including hospitals and Primary Healthcare units) was the most frequent possible source of infection (about half of the cases). At the same time, in the present study, the minor source of infection was the community (19.4%) which agreed with the studies carried out in Spain ^37^ and the Netherlands ^38^, which offered evidence of a pertinent role of the community as a source of transmission of infection to HCWs. These findings could imply that household contacts could have a substantial role in the COVID-19 disease in HCWs, primarily due to the quick community spread of the virus. The additional explanation might be that the infection from symptomless carriers, considering that nearly half of healthcare workers infected with COVID-19 were symptomless during the screenings ^28^. 

In the current study, most self-reported COVID-19 cases (about 71%) were probable cases, confirmed cases represented about 23%, and a tiny percentage of cases were asymptomatic (2.6%). In COVID-19, the severe cases represent a minority e.g. 14.8% of the cases among healthcare workers in China were categorized as severe or critical ^27^. Severe cases are not adequately reported due to their high mortality.

In the present study, the most frequent symptoms were fatigue/general weakness (74.1%) and a sore throat (72%), followed by headaches and myalgia, and the least frequent was mental confusion (7.8%). In the systematic review and meta-analysis of COVID-19 infection in HCWs, the frequently reported symptoms were fever (57%) and dry cough (57%), followed by malaise (43%) and myalgia (48%) among symptomatic HCWs ^28^. Similarly, the five most common symptoms among infected HCWs in China were fever (60.9%), myalgia or fatigue (60.0%), cough (56.4%), sore throat (50%), and muscle pain (45.5%) ^33^. Fatigue, ageusia or anosmia, and hoarse voice were persistent symptoms among frontline HCWs in the study carried out in the UK and the USA ^5^.

Positive chest computed tomography scan (C.T scan) findings were found to be 81.5% among the studied COVID-19 cases. This result agrees with that reported in China ^39^, where 86.2% of C.T. scans performed at the time of admission showed abnormal results.

## Study limitations

Selection bias was a potential limitation to the study due to non-random sampling of the study population since the use of online surveys as a data collection tool is limited to has selectivity among certain socioeconomic and age groups familiar with this type of online tool. The possibility of overestimation cannot be excluded due to the self-reported nature of the study. The severity of COVID-19 and mortality were not assessed in this study. 

## Conclusions

The self-reported incidence of COVID-19 infection among studied Egyptian physicians is 65.4%. Smoking, being a frontline physician, having contact with a COVID-19 case, and working for less than 10 years are all factors associated with an increased risk of infection. The strict application of preventive measures for all HCWs, including adequate PPE supplies and their proper use in the workplace, enables workers to keep a safe distance to avoid overcrowding and infection transmission. Periodic screening for COVID-19 for early detection and isolation of infected HCWs must be provided together with effective vaccination. Particular attention should be given to smokers, frontline doctors, contacts with COVID-19 cases, and doctors with short working duration. Implementation of an electronic registry for documentation and registration of both general COVID-19 cases and HCW cases will help monitor the magnitude of COVID-19 infection over time and affect different intervention measures. 

## Data availability

### Underlying data

Harvard Dataverse, V1: Self-reported COVID-19 among physicians: An Egyptian online study during the pandemic https://doi.org/10.7910/DVN/BVSLG2 ^40^

The project contains the following underlying data:

- File name: Covid dr incid. short.tab
- File description: 30 Variables, 355 Observations

### Extended data

Harvard Dataverse, V1: Self-reported COVID-19 among physicians: An Egyptian online study during the pandemic: Online Questionnaire 2021 https://doi.org/10.7910/DVN/BVSLG2 ^40^

- This project contains the following extended data:
- Questionnaire (in English and the introduction in original language)

Data are available under the terms of the Creative Commons Zero "No rights reserved" data waiver (CC0 1.0 Public domain dedication).
