# Supplementary material for: Self-reported COVID-19 among physicians: An Egyptian online study during the pandemic
Source: F1000Res. 2021 Aug 10;10:785. [Version 1] doi: 10.12688/f1000research.53931.1 (PMC8447048; doi:10.12688/f1000research.53931.1)
Supplement: Peer review history

# Peer review: Reviewer response for version 1

**DOI:** 10.5256/f1000research.57366.r91602

**Published:** 2021-09-16

**Authors:** Ibrahim Ali Kabbash

**Affiliations:** 1 Public Health and Community Medicine Department, Faculty of Medicine, Tanta University, Tanta, Egypt

1. The authors did not give an operational definition of diagnosis of COVID-19 among studied participants as suspected, probable, or confirmed. It seems that the diagnosis was very subjective. This should be mentioned as one of the limitations of the study.
2. Volunteer bias is suspected as those who had suspected signs might be more likely to respond to the questionnaire than those who did not.
3. Statistical analysis and multivariate analysis are of limited value, as the sample was not chosen randomly. This will affect seriously the ability to generalize data.
4. Results of this study should be considered with caution to the multiple limitations regarding sampling, the sample size that cannot represent national figures, and the subjectivity of self-reported data.

Is the work clearly and accurately presented and does it cite the current literature?

Yes

If applicable, is the statistical analysis and its interpretation appropriate?

Yes

Are all the source data underlying the results available to ensure full reproducibility?

Yes

Is the study design appropriate and is the work technically sound?

Yes

Are the conclusions drawn adequately supported by the results?

Yes

Are sufficient details of methods and analysis provided to allow replication by others?

Yes

Reviewer Expertise:

NA

I confirm that I have read this submission and believe that I have an appropriate level of expertise to confirm that it is of an acceptable scientific standard.

# Peer review: Reviewer response for version 1

**DOI:** 10.5256/f1000research.57366.r91640

**Published:** 2021-09-15

**Authors:** Indranil Saha

**Affiliations:** 1 Department of Community Medicine, IQ City Medical College and Narayana Multispeciality Hospital, Durgapur, West Bengal, India

**The article is nicely written but it needs the following clarifications:**

1. The study period is written as December to January, but what about the reference period? It has to be mentioned as it is written as a longitudinal study. Again, study subjects responded only once, then how can it be a longitudinal study.
2. How the sample size was decided as 323? The assumptions are written, whether these assumptions were put in a formula for longitudinal study? Please clarify.
3. Table 1, 2, 3, 4: subheading is missing.
4. Table 1: Please correct the title of the table. Whether incidence would be mentioned or not?
5. What was the operational definition of “probable cases” and “confirmed cases”? Please mention.
6. How much variation of dependent variable could be explained by the independent variables from the logistic model? Please clarify.
7. Discussion: 1 ^st^ line – please edit

Is the work clearly and accurately presented and does it cite the current literature?

Yes

If applicable, is the statistical analysis and its interpretation appropriate?

Yes

Are all the source data underlying the results available to ensure full reproducibility?

Yes

Is the study design appropriate and is the work technically sound?

Yes

Are the conclusions drawn adequately supported by the results?

Yes

Are sufficient details of methods and analysis provided to allow replication by others?

Partly

Reviewer Expertise:

Communicable and non-communicable diseases

I confirm that I have read this submission and believe that I have an appropriate level of expertise to confirm that it is of an acceptable scientific standard.
